# Investigation of user behavior on social networking sites

**DOI:** 10.1371/journal.pone.0169693

**Published:** 2017-02-02

**Authors:** Hajra Waheed, Maria Anjum, Mariam Rehman, Amina Khawaja

**Affiliations:** 1 Department of Computer Science, Lahore College for Women University, Lahore, Pakistan; 2 Department of Psychology, Lahore College for Women University, Lahore, Pakistan; West Virginia University, UNITED STATES

## Abstract

Social networking sites (SNS) are used for social and professional interaction with people. SNS popularity has encouraged researchers to analyze the relationship of activities performed on SNS with user behavior. In doing so, the term “user behavior” is rather used ambiguously with different interpretations, which makes it difficult to identify studies on user behavior in relation to SNS. This phenomenon has encouraged this thorough research on the characteristics of user behavior being discussed in the literature. Therefore, in this study, we aim to identify, analyze, and classify the characteristics associated with user behavior to answer the research questions designed to conduct this research. A mapping study (also called scoping study), which is a type of systematic literature review, is employed to identify potential studies from digital databases through a developed protocol. Thematic analysis is carried out for the classification of user behavior. We identified 116 primary studies for full analysis. This study found seven characteristics associated with behavior that have direct influence on SNS use and nine factors that have an indirect effect. All studies were conducted largely under seven areas that set the context of these studies. Findings show that the research on SNS is still in its early stage. The range of topics covered in the analyzed studies is quite expansive, although the depth in terms of number of studies under each topic is quite limited. This study reports that activities performed on SNS are either associated with user behavior or reflect personality characteristics. The findings of this study could be used by practitioners to evaluate their SNS platforms and develop more user-centered applications. These studies can also help organizations to understand better the needs of their employees.

## Introduction

Social networking sites (SNS) are virtual communities that allow people to interact and connect with each other by making profiles and sharing and uploading of photos and statuses [1, 2]. The use of SNS is not only a mere trend, but has become part of every person’s life [3]. This is evidently shown by the many millions of users of SNS, and with every passing day, the number is increasing [4, 5]. In the last decade, many SNSs have emerged popular among Facebook, Twitter, MySpace, Google Plus, and Flicker [6, 7]. This trend has lead researchers to conduct studies on SNS and its relation to user behavior. Various studies have been conducted to study user behavior under different contexts that include, and not limited to, privacy, trust, information sharing, gender, and geographical distances. Some studies have focused on the motivational factors of users to use SNS. Their identified factors include social consciousness, acquiring social presence [8], avoiding loneliness [2], leisure and entertainment [9, 10], acquiring the feeling of connectedness [9, 11, 12], extending ones social circle [13], and to voice ones opinion [14, 15].

Studies on SNS and user behavior have been conducted in the context of intensity of use, privacy concerns [16, 17], disclosure rates [18, 19], personality traits [20, 21], cultural norms [22], self-presentation [23], gender differences [24], age differences, and self-esteem [16, 25]. All these aspects cumulatively affect user behavior. However, in literature, each study has focused on one particular aspect. Therefore, a study providing a holistic view of influential characteristics of user behavior on SNS use is missing. In addition, a thorough review on the type of user behavior being studied by SNS research community is not available. This type of study can help SNS providers to identify features that can either be added or improved in the existing SNS. Furthermore, these studies could be helpful to understand how different characteristics of behavior could affect the virtual presence of a user. These studies could also help academic institutes and professional organizations to understand better the behavior of their employees.

In this research, we have conducted a systematic mapping study to investigate the level of evidence available in published literature on the relationship of activities performed on SNS and user behavior.

The succeeding section describes the research method employed. Section 3 explains the process of identification of primary studies. Section 4 presents data extraction. Section 5 provides analysis and discussion, while in Section 6, threats to validity are discussed. Finally, Section 7 concludes this research.

## Research Method

**Fig 1:**
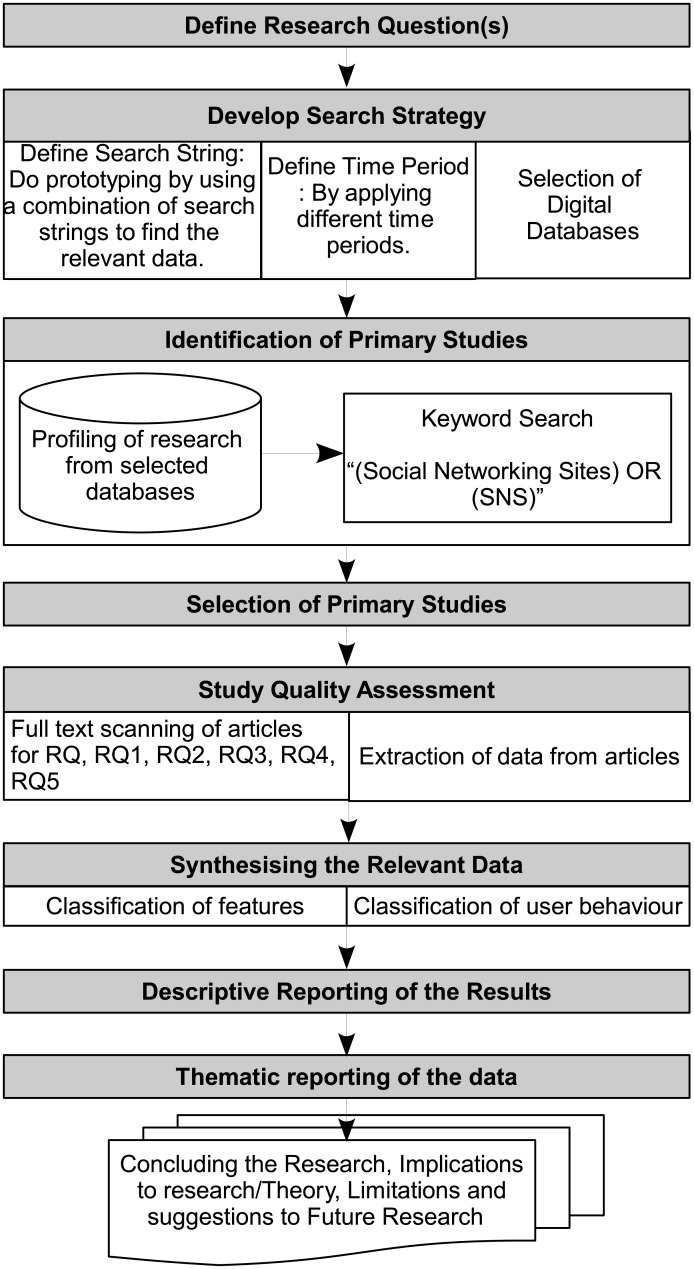
Research process.

This research has employed a mapping study (also called scoping study), which is a type of systematic review. Mapping study is a more open form of systematic literature review (SLR), which is conducted on an expansive topic to provide an extensive and in-depth overview of an area to assess the quality and quantity of the evidence available for that topic. Furthermore, it identifies gaps in the primary studies to determine where new studies might be conducted [26]. In this research, the process of conducting the mapping study is based on the guidelines available in Kitchenham et al. [27]. Fig 1 provides details on the research process adopted in this research. The primary studies were searched from the selected databases, which are presented in the next section. The studies were identified by applying inclusion and exclusion criteria. The data extraction was performed and synthesis was carried out for classification of user behavior. Finally, findings are provided to address research questions.

The overarching research question for this research was *What characteristics of user behaviour have been discussed by the social networking sites research community?*. Other associated research questions are:

- RQ 1: What is the trend in conducting research on social networking sites?
- RQ 2: Where primary studies are being published?
- RQ 3: What countries are being selected as a case study?
- RQ 4: What type of social networking sites are being used by participants?
- RQ 5: Which research method is largely adopted by studies?
- RQ 6: In which context studies on SNS user behaviour are being conducted?

A thematic analysis is carried out on the primary studies to answer these research questions. Studies that have used human participation and have employed any combination of research methods, such as experiment, survey, and interviews, are being considered for this research. Studies that use automatic data extraction from user profiles are not considered suitable for this research because involvement of participants bring their own understanding of particular actions and provide context, which is missing in automatic data extraction.

### Search Strategy

The search strategy designed to conduct this research is discussed below.

#### Search String

To find primary studies, different combinations of search strings were designed that are available in Table 1. The search was conducted using these queries and a comparison was made on the initial results. After careful analysis of the results, the search string that brought relevant and maximum results was selected.

**Table 1:** Search Strings.

Initial Strings	(Social Networking Sites) OR (SNS) OR ((Social Networking Sites) AND (Facebook)) OR ((Social Networking Sites) AND (Twitter)) OR ((Social Networking Sites) AND (LinkedIn)) OR ((Social Networking Sites) AND (Behavioral Patterns)) OR ((Social Networking Sites) AND (Usage Patterns))
Final String	(Social Networking Sites) OR (SNS)

The results show that other queries did not bring significant dataset. The final selection of string brought all the results displayed by other queries and the ones that did not appear in their result set. Furthermore, the strings that explicitly focused on the usage and behavioral patterns did not bring sufficient results because in many papers, the word behavior was not mentioned despite being discussed in the paper.

#### Time Period

The time period selected for this research was from 2005 to 2013. We found that the research on SNS is quite new and no paper appeared before 2005. The result was explicitly verified against the years 2000 to 2004 to check if any paper is missed. However, we did not find any publication on social networking sites before 2005.

#### Selection of the Electronic Databases

To find primary studies, three databases were selected that include:

- Science Direct
- ACM Digital Library
- IEEExplore

These three databases were selected because they are reliable and the studies published are peer reviewed, which provides a quality check of primary studies. Kitchenham et al. (2009) mentioned these databases for exhaustive search in software engineering. Furthermore, these databases are known to include empirical studies and literature surveys. All results that appear in these databases were taken into consideration.

#### Selection of Primary Studies

The studies were selected according to the inclusion and exclusion criteria defined in Table 2.

**Table 2:** Inclusion and exclusion criteria.

Inclusion Criteria	Search String should appear in title or abstract of the paper. The paper should be in English. The paper should discuss the behavior of SNS users. Research method used must fall under the category of survey, interview, and experiment. The papers must have human participation.
Exclusion Criteria	Keynotes, abstracts and extended abstracts, conference panels and summaries, magazine features, short papers, and work in progress papers were excluded. Papers published on topics such as politics, religion, healthcare, and drugs were excluded.

## Identification of Primary Studies

The search was conducted on the selected databases by using the final search string on titles and abstracts of primary studies. The results obtained from each database are shown in Table 3.

**Table 3:** Identified primary studies from databases.

Databases	Papers Found
Science Direct	224
ACM	767
IEEE	1690
Total	2681

**Fig 2:**
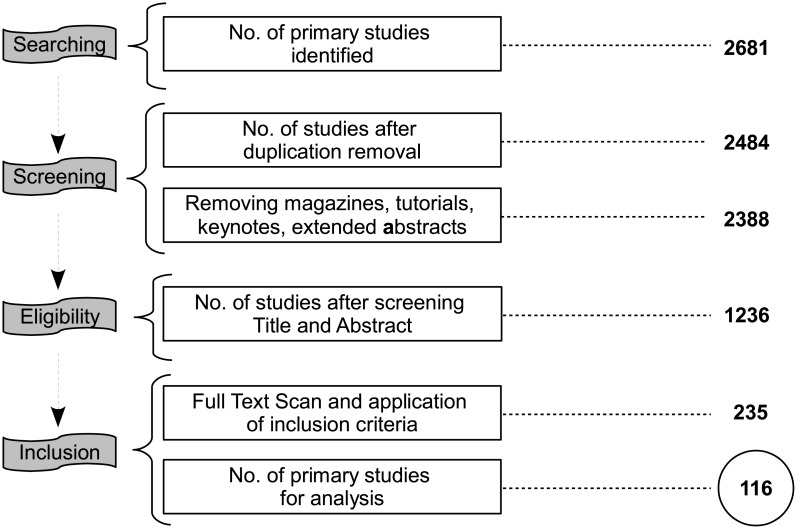
Primary studies selection process.

The selection of primary studies was carried out by the following four distinct steps presented Fig 2.

**Step 1: Searching:** We identified 2681 studies that contained search string in their titles or abstracts. The criteria that the search string must appear in the title or abstract was followed strictly.
**Step 2: Screening:** The papers identified in the first phase were screened to remove duplications that excluded 197 studies. The exclusion criterion was applied on 2484 papers that reduced the total count to 2388 papers. At this point, we excluded papers that came under the category of extended abstracts, keynotes, dissertation papers, and papers in other languages, such as French and Chinese.
**Step 3: Eligibility:** In this phase, the titles and abstracts of 2388 papers were analyzed to determine their relevance that made us exclude 1152 papers. A total count of 1236 studies comprises the final phase.
**Step 4: Inclusion:** We examine the full text of 1236 studies to identify papers related to user behavior. By applying the inclusion criteria, 235 papers were selected for full-text scanning.

The papers that primarily focus on topics such as information dissemination, algorithms, recommendation systems, security attacks algorithms, privacy concerns algorithms, clustering, graphs, developing search engines related to SNS, SNS traffic jams, detecting spammers and attackers by clustering algorithms, community detection algorithms, extraction methods involving algorithmic techniques, infrastructures dealing with networking and routing information, algorithms for the same detection, ranking algorithms, prediction algorithms to predict the future of a user or relationship between two friends on SNS, machine learning approach to detect patterns or cyber bullying, profit models for SNS, and mathematical models for SNS were excluded. Furthermore, papers that mainly focus on religion, politics, marketing, healthcare, diseases and disorders, and effects of drugs in SNS users were also excluded.

During the second round of full text analysis, we excluded papers that focused on measuring SNS effects in the context that was not within the scope of our research, such as studies on SNS usage as an e-learning tool, to increase spectatorship on an event or game, to analyze reality television viewers, to improve business, and the use of customized SNS (such as Beehive), which was not publicly accessible.

After carefully scanning the complete text, 116 studies were left to be included for analysis. The detail of the selected studies in each database is shown in Table 4.

**Table 4:** Studies found and included in the mapping study.

Databases	Studies Found	Studies Selected	Included
Science Direct	224	82	46
ACM	767	75	52
IEEE	1690	79	48
**Total**	2681	235	116

## Data Extraction

In this phase, a thorough examination of the 116 studies was performed. The data was organized by tabulating in spreadsheets. A unique paper ID was assigned to each study. The data was coded by extracting information about the author, year of publication, source database, research method employed, area of research conducted, participant details, study scope and context, the characteristics that define behavior, and study findings. The detail of the assessment criteria is described in Table 5.

**Table 5:** Content assessment criteria.

Data extraction elements	Description
Type of Study	Journal and Conference Papers
Year of Study	Year primary studies published
Research Method	Survey, Experiment, Interview
Study Focus	Motivation and objectives, hypothesis, research questions, description of the context, application domain of the study, features in influencing user behavior, user behavior characteristics, any other features studied in relation to user behavior, discussion, and findings.

## Analysis and Discussion

The thematic analysis was carried out on the final dataset of 116 studies. The study areas were focused to identify the context in which user behavior was discussed. The content was analyzed by two analysts and reviewed by two checkers to identify if any inconsistency exists. This method of analysis in the mapping study is supported by previous studies of Kitchenham et al. [27].

### Yearly publications

**Fig 3:**
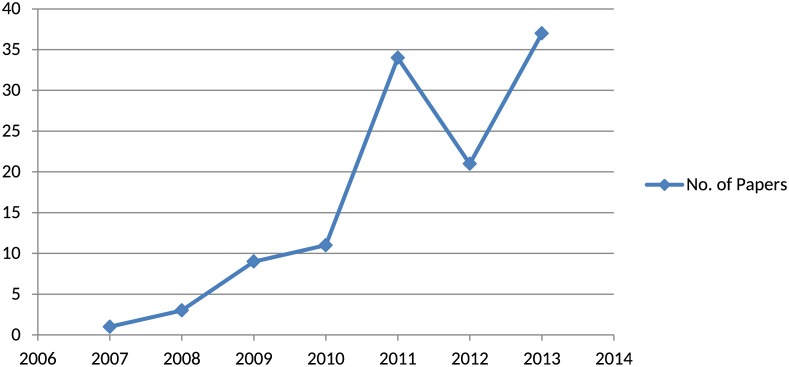
Number of papers by year of publication.

To answer RQ 1, the year of publication of each primary study was tabulated. We identify that SNS is an emerging area and the studies published on this topic begin to appear in databases from the year 2005. In Fig 3, a graph is presented to show the trend of publication over the years. It is quite evident that this area is gaining more interest and the number of publication is increasing over time. There is a slight decline in the year 2012, which could be attributed to studies published in this year that did not meet our selection criteria. However, in the year 2013, publication increased, which signifies the interest in this area.

### Types of publication

To answer RQ 2, we analyzed the sources of each study where they were published. From the selected studies, 70 studies were published in conferences and 46 in Journals. The name of each journal and the number of studies found are shown in Table 6. The details of each study are listed in Tables A-I in S1 Appendix.

**Table 6:** Journal Names and no. of Collated Studies.

Journal Names	No.of Studies
Computers in Human Behavior	36
Information and Management	1
Decision support System	3
International Journal of Human Computer Studies	1
Journal of Computing Sciences in Colleges	2
Journal of Theoretical and Applied Electronic Commerce Research	2
IEEE Transactions on Engineering Management	1

### Geo-spatial coverage for case studies

**Fig 4:**
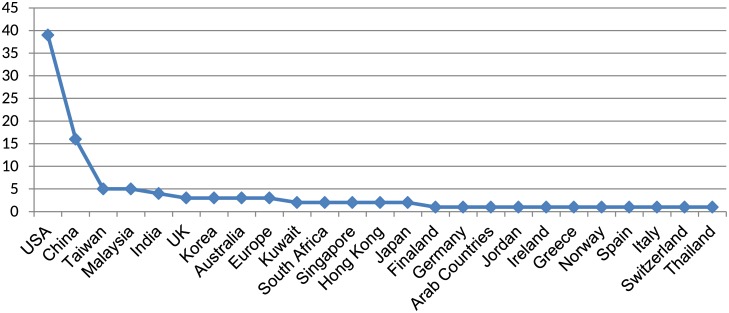
Case studies from different Countries.

RQ 3 is addressed by analyzing the background of participants. Some studies explicitly mentioned the countries selected for the study. However, there are studies where this information was missing; therefore, we analyzed the background of participants. In some cases, studies made a comparison of user behavior in two different countries. Furthermore, there are studies that took participants from Asia, Europe, and Arab world instead of focusing on a single country alone. A list of studies with their participants for each country is shown in Fig 4. The figure provides a number of studies and their target country. Studies that did not provide information about their participants and recruited them through Amazon Mechanical Turk or through advertisements and e-mails are not included. These studies did not focus on a specific country and emphasized on user behavior without considering their background or culture. The highest number of participants was 39 and the country with most participants was the USA.

### Types of SNS used

For RQ4, SNSs discussed in the studies were focused and a list was prepared. In Table 7, the names of the SNS that each study focused on are presented. The SNS used by participants fall under different categories. There are SNS-specific to a particular region, such as Tuenti for Spain, Renren, Kaixiniare for China, and StudiVZ for Germany and Austria.

**Table 7:** Social networking sites discussed in primary studies.

Social Networking Site (s)	No.of Studies
Facebook	56, 7*, 14+
Facebook & Renren	1
Facebook, Renren & Kaixin001	1
Facebook & LinkedIn	1
Facebook & Twitter	1
Facebook & MySpace	1
Facebook, MySpace & Twitter	1
Facebook & Bebo	1
Facebook & StudiVZ	1
Facebook, Twitter, LinkedIn, Orkut, YouTube & Flickr	1
Facebook, Hi5, You Tube, Twitter & MySpace	1
myYearbook, Facebook, twitter & MySpace	1
Siena Weibo & Renren	1
Kaixin	1, 2+
Tuenti	1
MySpace	1, 3+
Flickr	1
Renren	5, 5+
XiaoNei & KaiXin	1
Qzone, Renren & Sina Weibo.	1
renren, kaixin001, pengyou, jiayuan, baihe & sina friends	1

^+^ Symbolizes the overlapping studies that have focused on more than one SNS.

* represents that the study did not specifically focus on Facebook; however, it was found to be the most popular SNS used within the sample population.

**Fig 5:**
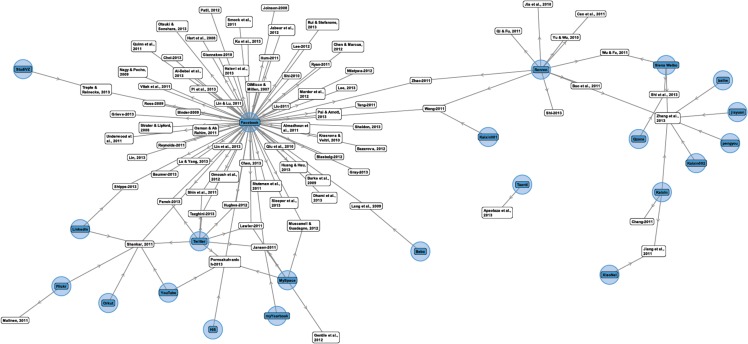
Clusters of Social Networking Sites and associated Studies.

SNSs that do not belong to any particular culture and are popular around the globe include Facebook and Twitter. Facebook is the most prevalent SNS where a wide number of studies have been conducted. The statistics from studies represent that Facebook is the most popular SNS currently in use. Studies that do not focus on any particular SNS although they discussed user behavior were not listed in this table. In Fig 5, a cluster view of these studies is provided. The cluster around Facebook is thicker than any other SNS.

### Research method used

RQ 5 is addressed by analyzing the research method employed by each study. Table 8 provides detail on the research method and the number of studies that fall under each method. There are studies that used a combination of all three methods. It is evident from the table that surveys are the highly employed tool for these studies.

**Table 8:** Research methods used.

Research Method	No. of Studies
Survey	92
Interview	10
Experiment	8
Survey and Interview	4
Survey and Experiment	1
Interview and Experiment	1

### Context of studies

**Fig 6:**
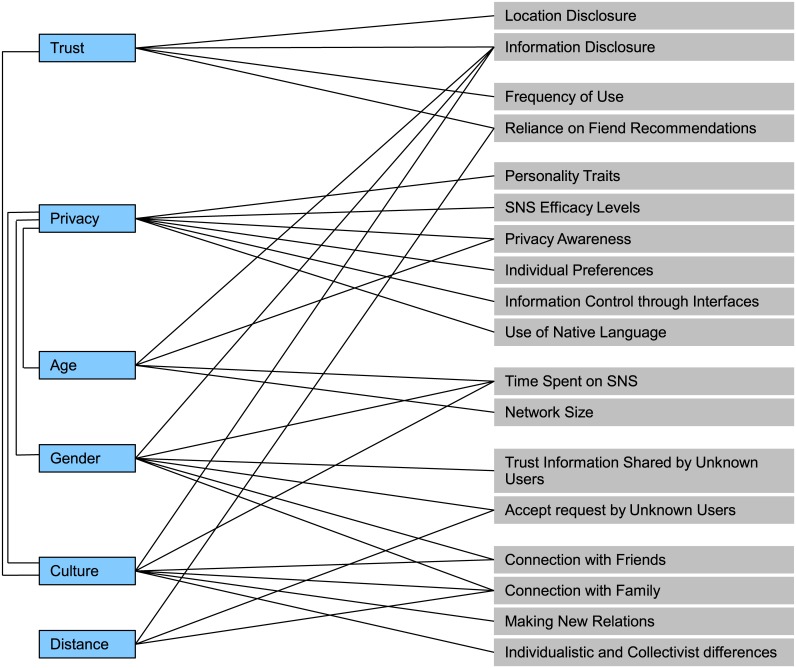
Context of studies.

The factors that set the context of studies were identified to answer RQ 6, which include trust, privacy, age, culture, gender, distance, and information sharing. In Fig 6 the context in which these studies are conducted is presented.

**Fig 7:**
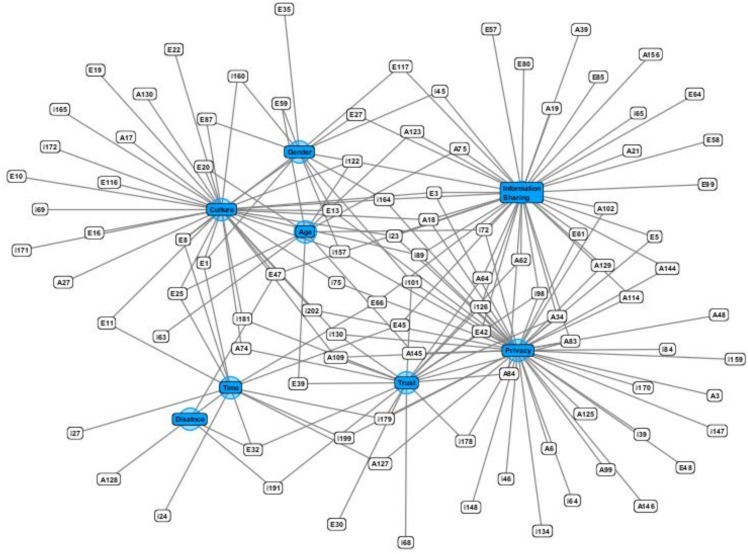
Clusters of social networking sites and their associated studies.

In S1 Appendix, the studies from where these factors have been derived are listed in Table A-I. Some studies included more than one factor and were listed twice. In Fig 7, clusters formed around each factor and overlapping of these is shown. From the Figure, it is clear that user behavior is mostly discussed in the context of privacy, information sharing, and culture. The users are more concerned about their privacy either provided through SNS interfaces or self-employed.

### Findings, analysis and discussion on identified behavioral characteristics of SNS users

To answer the overarching research questions posed early in the paper, we identified features that are related to user behavior from 116 studies summarized in Tables 9 and 10. The description of these features is extracted and their context is explained. There are features that do not directly come under user behavior; however, they contribute in forming user behavior, such as personality traits. These features cannot be termed as a form of behavior; however, together with SNS use, they help to differentiate user behaviors.

**Table 9:** SNS user behavior characteristics identified cont.

Behavior Characteristics	Details	No. of Studies
Frequency of use	Usage in terms of time spent on SNS, frequency of visits, posting information, frequently updating statuses.	55
Information Control	Information control through interface features to maintain privacy.	3
Self-disclosure/ Self Projection	The extent and type of information disclosed on SNS. User’s disclosure is related to their awareness and concerns about privacy.	24
Self Censorship/ Self Awareness	The extent and type of information disclosed and revealed by a user and usage of privacy settings for one’s own convenience. Self awareness in terms of user’s knowledge about the privacy settings and policies and how users gain an insight into other user’s life through SNS.	10
Self-efficacy	The level of shared information that one controls and awareness about privacy settings and features.	2
Ease of use	Time and effort required to learn and use SNS interface.	3
Self-Presentation/ Identity Management/ Self Orientation	Maintaining self image (such as presenting oneself in an attractive manner, sharing appropriate information for maintaining impressions on others and telling lies to maintain or project ones’ identity), status symbol, sharing information (such as pictures) with the notion of how others perceive them.	27
Social Affiliation/ Belonging	Feeling an integral part of the social circle, belonging.	1
Social Connection	Making friends, keeping in-touch with old friends, developing new relationships, and maintaining existing relations.	7
Social Capital	Maintaining existing relationships, stronger ties (close family and friends), developing new relations and weaker ties. Support, trust, reliance, and information or knowledge derived from these relationships.	12
Personality Traits	Personality traits make users behave differently on SNS. Five traits, namely, extroversion, agreeableness, narcissism, neuroticism, and conscientiousness are discussed extensively with the usage differences in (relaxation, killing time, connecting with friends and family, status symbol, social influence, social interaction), information sharing, privacy, and disclosure	18
Attitude	Positive or negative attitude towards SNS determines its usage. A positive attitude towards SNS increases its use.	13
Gratifications	Satisfaction and pleasure derived from SNS use. Gaining recognition, making one’s image, status symbol, social interaction and communication, connecting with friends and family, making new contacts, are the gratifications derived from SNS use.	10
Relaxation/ Hedonic/ Involvement/ Enjoyment/ Entertainment	SNS usage for enjoyment, leisure, entertainment and relaxation. It also includes involvement in terms of how much users are immersed in SNS that they forget their surroundings.	17
Self Esteem	Posting comments on SNS increase with high self-esteem. Frequent participation for gaining recognition and acknowledgment, feeling important, valuable and increasing the sense of self-worth.	7
Social Presence	Mitigating loneliness, sense of warmth, and feeling of being valued and worthy.	8
Social Influence/ Social Fetter/ Peer Pressure/ Family Pressure	SSubjective norms, family or peer pressure that motivate or demotivate SNS usage and forms an attitude (good/bad) towards SNS.	12
Social Norms	Norms or rules in one social circle will lead to ones participation and information sharing on SNS. Social norms determine user behavior, such as level of participation (usage and network size).	3
Surveillance/ Social Investigation	Investigating profiles of friends and unknown to gain insight into their lives especially in relationships.	3
Regrets/ Tension/ Anxiety	Regret after posting/sharing emotional stuff and its consequences in the form of pressure from the network.	5

**Table 10:** SNS user behavior characteristics identified.

Emotion	Feeling comfortable to express sentiments and feelings on SNS than face-to-face.	2
Boredom	Passing time, relieving boredom, killing time on SNS.	4
Reciprocity	Active participation on SNS from friends makes the user contribute and connect with them in the same pace.	2
Shyness/social boldness	Shyness, hesitant to share and impact on disclosure.	3
Self control	Using SNS in a controlled manner and not addicted to it (i.e., not depending on it too much to use it every day).	2

From the features listed in Tables 9 and 10, we separated those that come under the category of user behavior. As various terms were used to represent a single concept, we grouped the relevant terms and allocated them with identifiers that are more suitable to represent the concept. Terms are the features used in studies of user behavior. Identifiers are the labels assigned by us to group these terms. We also provided a concise description of each of these identifiers. Some identifiers are taken from the vocabulary of social psychology, where appropriate. In Table 11, classification of user behavior is provided.

**Table 11:** Classification of User Behavior.

Identifiers	Description	Terms
Social Investigation	To explore, search, and examine activities of other users on SNS.	Surveillance, social investigation, social surfing, browsing
Social Affiliation	Bonding with others (friends, family, and peers) on SNS, developing and maintaining new ties. Associating with the social circle on SNS and feeling an integral part of it. Resources derived from SNS bonds and connections.	Social interaction, social communication, social connection, belonging, involvement, social affiliation, social capital, stronger ties, weaker ties
Frequency of use	Time spent on SNS and the extent of activities performed on it.	Frequency of visits, frequency of updating statuses
Information Control	Managing information through access control features on SNS and controlling the level of information disclosed on SNS.	Privacy interface features, self-censorship, self-awareness, self-Efficacy, self-disclosure, self-projection
Self Orientation	Creating favorable impressions on SNS for presenting the desired image of oneself.	Self-presentation, identity management, self-orientation
Reciprocity	Participating on SNS in the same frequency as their SNS contacts or friends.	Reciprocity
Social Boldness	Hesitation in contributing and participating on SNS.	Shyness

Social investigation, social affiliation, frequency of use, information control, self-orientation, reciprocity and social boldness are the user behavior characteristics that directly affect SNS use.

- Social investigation involves exploring and searching about others on SNS and trying to keep an eye on them. This behavior is common in relationships or when users are away from home. In addition, geographical distances play a key role in this behavior.
- Interactions, communication, and bonds formed on SNS are termed as social affiliation. Each individual has his/her own pace of participating in SNS, which ultimately determine the intensity of interactions and bonds formed.
- The frequency of visiting, time spent on, and the intensity of use of SNS are also determinants of the user behavior.
- Information control concatenates the intensity of disclosure, concerns related to sharing content on SNS, and using access control features to mitigate these concerns.
- While using SNS, users want to create the desired image of themselves, and to satisfy this desire, they manage their impressions by presenting themselves in a favorable manner. The most common example of this is uploading pictures especially taken for posting on SNS. This behavior falls under self-orientation.
- Social influence is another factor influencing behavior; with family, friends, peers, and other contacts actively participating on SNS, a user feels a need to reciprocate in the same manner. This motivates them to participate more, hence impacting behavior in terms of usage.
- Social boldness is a term derived from Social psychology and deals with the hesitant nature of users to participate on SNS. Shyness is an important determinant of this behavior. People who are shy participate less and rarely post or update statuses.

As mentioned previously, there are features that cannot directly be characterized as user behavior; however, they have an influence on user behavior while surfing SNS. We classified these features and description for each identifier is provided in Table 12.

**Table 12:** Features having impact on user behavior.

Features	Description	Terms
Ease of use	Skill or effort required to operate SNS. Ease of use	
Gratifications	Sentiments of pleasure derived from using SNS. Flow experience while using SNS.	Gratifications, relaxation, hedonic, enjoyment, entertainment
Personality Traits	Individual characteristics that form user behavior on SNS and differentiates it from others. Positive or negative attitude towards SNS that impacts its usage.	AAttitude, personality traits w.r.t. Big Five extroversion, agreeableness, narcissism, neuroticism, and conscientiousness
Self esteem	Frequent SNS participation for gaining recognition and acknowledgment to increase self-worth. Being socially present in SNS interactions and activities that generate feelings of worthiness.	Self-esteem, social presence
Social influence	Family, friends, or peer influence that impacts SNS usage. Rules and customs in ones social circle that determine and impact SNS usage.	Social influence, social fetter, peer pressure, family pressure, social norm
Regret	Consequences of posting inappropriate content on SNS.	Regret/ tension/ anxiety
Emotion	Expressing one’s sentiments on SNS easily and openly.	Emotion
Boredom	Using or participating on SNS to pass the time, or relieve boredom and dullness.	Boredom
Self-control	Capacity to control SNS use and participation.	Self control

- Ease of use determines the skill to operate and use SNS. If the SNS are easy to operate and less effort is required to learn it, then users will consider it as a motivational factor for its usage. Therefore, ease of use itself cannot be categorized as a behavior; however, it does influence SNS use.
- Gratifications and hedonic are termed as one feature because they both deal with sentiments of pleasure. Users who enjoy SNS and are contented with it are more apt to use it later, which leads to greater participation.
- Personality traits are the individual characteristics that differentiate individuals from each other. In SNS, personality traits are one of the major determinants that predict user behavior. In personality traits, apart from the Big Five traits that are also termed as OCEAN (Openness to Experience, Conscientiousness, Extroversion, Agreeableness, and Neuroticism), another characteristic narcissism is also discussed. Narcissism is associated with the posting of pictures that are more attractive and perceived as more appealing to obtain rave reviews and comments [28]. Similarly, extroversion is linked with a larger network size [29]. Since a set of behavior is associated with each of these traits, personality traits do influence user behavior.
- Self-esteem is also a trait that has an effect on user behavior. It is defined in terms of self-evaluation and how high one regards him or herself. People having a low self-esteem broaden their social circle by communicating with people that they cannot interact in the offline world [25]. They try to manage their low esteem by frequent social interactions on SNS and having an extensive friend list [30].
- The social influence, which includes family, friends, peers, culture, and social norms, also have an impact on user behavior. Family and significant others participating heavily on SNS will be a motivational and influential drive to use and participate on SNS.
- Negative incidents associated with SNS use will dissuade a user from active participation, thereby influencing behavior and usage in a negative way. These negative incidents may be the consequences of posting an inappropriate stuff or due to harsh and vile comments.
- Some people find SNS more appropriate for venting out their emotions than expressing them face-to-face. This attitude positively impacts their usage and participation.
- Some users find SNS a useful tool for passing time or to relieve boredom. This attitude motivates them to use and participate on SNS whenever they feel a need to kill time.
- Self-control is the ability of a person to control his/her use of SNS. If an individual wants to control the addictive use of SNS, then this attitude will deter him/her from excessive use and will influence SNS user behavior.

From previous discussion, we can conclude that behavior cannot be measured directly. Instead, activities performed by the user on SNS helps in determining user behavior. Apart from behavior, there are various factors, including personality traits and others discussed previously, that influence user behavior and activities performed on SNS.

## Threats to Validity

Internal validity is considered as a valid main threat in the mapping study because it is a form of secondary study and does not involve human participation. Construct validity could be an issue; however, by following the guidelines provided by Kitchenham et al. (2007), which are now well established, this threat is no longer considered.

### Internal Validity

**Search String:** It is possible that we have missed papers that discussed the behavior of SNS users. However, we prototyped the search strings to identify relevant studies (the search string selection process is discussed in Section 3). Since we included all studies that contained the search string we consider it a minor threat.
**Search Coverage:** We have used three of the major databases (IEEE, ACM, and Science Direct) to conduct a search. Normal guidelines suggest four; however, we used the snowballing technique on references found in the studies and this did not point out any significant grouping of papers that we missed.
**Analysis of Papers:** SLR guidelines suggest that data extraction should be performed independently by two analysts or a single analyst and a checker to verify the percentage. The data extraction was performed by two authors. The analysis was conducted by authors and a psychology expert to check the level of agreement in the identified themes. The research covers papers from 2005 to October 2013. An informal check of subsequent publications on this topic does not suggest that there have been any significant changes.

## Conclusion

The purpose of this research was to explore the behavioral characteristics of SNS users. To find the evidence showing the extent of discussion on user behavior in the existing literature, we designed our overarching question as follows: What characteristics of user behavior have been discussed by the SNS research community? To answer the research question, mapping study research technique was employed, which is a type of systematic review. Since user behavior characteristics were not clearly defined in studies, a thematic analysis was performed to identify, analyze, and classify identified features. The behavioral characteristics identified include frequency of use, information control, social affiliation, self-orientation, reciprocity, social boldness, and social investigation. The context under which user behavior was discussed includes trust, privacy, age, culture, gender, information sharing, and distance. The factors that did not come under the category of behavior but have a direct or indirect influence on user behavior include social investigation, personality traits, social orientation, social influence, self-esteem, and information control.

The use of social media, especially social networking sites, is increased in the recent era. One of many reasons for this rapid adoption is the easy access and availability of various SNSs. Owing to the popularity of SNSs and their influence on users, this has become an important area of study for researchers. Current research has employed a range of research methods to evaluate user behavior in particular areas, such as privacy, information sharing, trust by considering different types of users, which are not limited to students, adults, teenagers, older adults, and professionals.

By conducting a mapping study, we identified that research on SNS is barely a decade old and there is no evidence available before 2005. Even studies published in 2005 were not relevant, although the search string (SNS) did appear in their titles or abstracts. The broad coverage of topics in these studies reflect an increased interest in this area; however, the depth in terms of similar studies is insufficient. This insufficiency could be attributed to the still emerging research on SNS. Out of 116 studies identified for full analysis, 70 were published in conferences and 40 studies were published in journals. The users participated in these studies had backgrounds from different countries, such as China, Mexico, Malaysia, Singapore, Thailand, America, Europe, Arab countries and others. The highest number of studies was conducted in America. The most popular SNS among participants was Facebook, although some region specific SNS, such as Chinese SNS Renren, were also found popular among their community.

In terms of research method employed by primary studies, we identified that surveys (both questionnaire and interviews) are the highly employed research method for determining and evaluating the behavior of SNS users. In some studies, multi-method techniques were employed; however, their number is quite small.

Under the scope of this research, we did not find evidence where characteristics of user behavior are fully considered in a single study. In addition, the term “user behavior” was used quite ambiguously. Some studies used this term without providing further details, while some studies did not use this term, although their work was closely related to the evaluation of certain characteristics of user behavior.

This study contributes to the theory by systematically analyzing evidence from literature and by providing an integrated view of user behavior. The study also provides a classification of user behavior that can help researchers to conduct a detailed study on the relationships of these behaviors and their effect on the SNS users. Furthermore, user behavior and activities performed on SNS could be studied in relationship to identified behavioral characteristics, and a comparison among different groups of SNS users could be performed.

The research findings could help practitioners in introducing improvements in existing SNS platforms by understanding user behavior and its relation to SNS use. By considering the elements of social psychology in the existing SNS, it can help to develop more user-centered applications. In addition, these studies could help academic institutes and professional organizations to better understand their employees and their needs.

This research is conducted under the scope of the criteria defined in the Research Method section. The threats to the validity of this research are being discussed in Section 7. A similar study with broad criteria could bring more evidence that could strengthen the findings of this study and contribute further details related to user behavior on social networking sites.

## Supporting Information

S1 Appendix Contains Tables A–I. (PDF)
