# Supplementary material for: Investigation of user behavior on social networking sites
Source: PLoS One. 2017 Feb 2;12(2):e0169693. doi: 10.1371/journal.pone.0169693 (PMC5289435; doi:10.1371/journal.pone.0169693)
Supplement: S1 Appendix — (PDF) [file pone.0169693.s001.pdf]

S1 Appendix

Table A. Details of Primary Study Publication Type, Year of Publication, Research Method, Participants and Duration

Paper Id	Year of Publication	Publication Type	Conference/Journal Name	Research Type	No. of Participants	Gender	Age
[1]	2011	Conference	13 International Conference on Human-Computer Interaction INTERACT	Survey	103	61 males, 42 females	18-44 years
[2]	2011	Conference	13th International Conference on Information Integration and Web-based Applications and Services	Survey	222	58 % females	13-35 years
[3]	2011	Conference	SIGCHI Conference on Human Factors in Computing Systems	Interview	27	17 males	earlu 20's early 30's
[4]	2011	Conference	iConference	Survey	34,514 teen and young adult users	NA	13-24 years
[5]	2012	Conference	iConference	Experiment	512	209 male, 303 female	NA
[6]	2013	Conference	SIGCHI Conference on Human Factors in Computing Systems	Survey	410	NA	19-76
[7]	2010	Conference	14th international academic MindTrek conference: envisioning future media environments	Survey	1st Study: 70 Facebook users, 2nd Study: 131 Facebook users	1st Study: 32 males and 38 females. 2nd Study: 59 males and 72 females	25.6
[8]	2012	Conference	Journal of Computing Sciences in Colleges	Survey	98 students	NA	NA
[9]	2011	Conference	SIGCHI Conference on Human Factors in Computing Systems	Survey	Total 114, 57 from China and 57 from USA	31 males and 26 females from each country	NA
[10]	2007	Conference	International ACM conference on Supporting group work	Survey, Interview	68 employees in Survey, 8 interviewed.	73% males and 27 % females.	NA
[11]	2013	Conference	Computer supported cooperative work	Interview	18	8 males, 10 females.	20 - 51
[12]	2011	Journal	Journal of Computing Sciences in Colleges	Survey	200	36.5 % males, 63.5 %, females	18.6
[13]	2013	Conference	22nd International conference on World Wide Web companion	Survey, Experiment	100	83 males, 17 females.	18 to 31years
[14]	2008	Conference	SIGCHI conference on Human Factors in Computing Systems	Survey	1st Study: 137 users, 2nd Study:241 users	1st Study: 53 males and 88 females, 2nd Study: 80 males, 161 females	1st Study: 26.9 years, 2nd Study: 25.97 years.

Table B. Details of Primary Study Publication Type, Year of Publication, Research Method, Participants and Duration

Paper Id	Year of Publication	Publication Type	Conference/Journal Name	Research Type	No. of Participants	Gender	Age
[15]	2012	Conference	ACM 2012 conference on Computer Supported Cooperative Work	Survey	1064	From USA: 343 males, 153 females, From China:99 females and 469 males	For USA:39.47 years, For China:30.77 years
[16]	2011	Conference	4th International Conference on Trust and trustworthy computing	Survey	924	NA	¿18
[17]	2012	Conference	ACM 2012 Conference on Computer Supported Cooperative Work	Experiment	220	NA	NA
[18]	2009	Conference	SIGCHI Conference on Human Factors in Computing Systems	Interview	7	NA	NA
[19]	2009	Conference	Fourth international conference on Communities and technologies	Survey	2435	69% male, 31% females	30-39
[20]	2011	Conference	4th International Conference on HCI International Online Communities and Social Computing	Interview	26	NA	NA
[21]	2011	Conference	Seventh Symposium on Usable Privacy and Security (SOUPS)	Survey	1st survey: 569 students 2nd Survey: 492, Interviews: 19	1st survey:117 males, 204 females, 2nd Survey:216 males, 276 females, Interviews: 10 males, 9 females	1st survey:Average 31 years, 2nd Survey:Average 28 years.
[22]	2009	Conference	8th International Conference on Mobile and Ubiquitous Multimedia	Interview	27	15 male,12 female	NA
[23]	2011	Conference	ACM 2011 conference on Computer supported cooperative work	Survey	30	NA	23.70
[24]	2008	Conference	5th Nordic conference on Human-computer interaction: building bridges	Survey	26	10 males, 16 females.	18 44
[25]	2013	Conference	5th International conference on HCI International Online Communities and Social Computing	Survey	1st Survey: 328 participants, 2nd Survey: 89 participants	NA	17-45
[26]	2012	Conference	SIGCHI Conference on Human Factors in Computing Systems	Interview	21	11 males and 10 females.	Average 36 years.

Table C. Details of Primary Study Publication Type, Year of Publication, Research Method, Participants and Duration

Paper Id	Year of Publication	Publication Type	Conference/Journal Name	Research Type	No. of Participants	Gender	Age
[27]	2013	Journal	Journal of theoretical and applied electronic commerce research	Survey	164	NA	NA
[28]	2013	Conference	Computer supported cooperative work	Survey	666	NA	NA
[29]	2013	Conference	5th Annual ACM Web Science Conference	Survey	Total: 1051 Users UK : 549, USA : 502	517 male, 534 female	NA
[30]	2010	Conference	7th International Conference on Advances in Computer Entertainment Technology	Experiment Ex	59	24 males, 35 females	Average 21 years
[31]	2011	Conference	Symposium on The role of design in UbiComp research & practice	Survey	80 users in survey,. 25 interviewed.	NA	NA
[32]	2009	Conference	SIGCHI Conference on Human Factors in Computing Systems	Survey	232	NA	22-27
[33]	2008	Conference	22nd British HCI Group Annual Conference on People and Computers	Experiment	18	6 male, 12 females	NA
[34]	2013	Journal	Journal of theoretical and applied electronic commerce research	Survey	221	NA	NA
[35]	2011	Conference	ACM SIGCOMM conference on Internet measurement conference	Survey	200	NA	NA
[36]	2013	Journal	Computers in Human Behavior	Survey	451	41% males, 59% females.	NA
[37]	2012	Journal	Computers in Human Behavior	Survey	265		18-24
[38]	2013	Journal	Computers in Human Behavior	Interview	24	NA	20-40
[39]	2011	Journal	Computers in Human Behavior	Survey	1324	NA	18-44
[40]	2011	Journal	Computers in Human Behavior	Survey	150	40% males, 60% females	18-23
[41]	2012	Journal	Computers in Human Behavior	Survey	340	41.2% males, 58.8% females	NA
[42]	2009	Journal	Computers in Human Behavior	Survey	97	15 men, 82 women	21.69
[43]	2012	Journal	Computers in Human Behavior	Survey	488	51% male, 49% females	18-60
[44]	2011	Journal	Computers in Human Behavior	Survey	676	NA	18-25

Table D. Details of Primary Study Publication Type, Year of Publication, Research Method, Participants and Duration

Paper Id	Year of Publication	Publication Type	Conference/Journal Name	Research Type	No. of Participants	Gender	Age
[45]	2013	Journal	Computers in Human Behavior	Survey	579	NA	Average 35.2
[46]	2010	Journal	Computers in Human Behavior	Survey	160	36% male, 64% females	13-16
[47]	2011	Journal	Computers in Human Behavior	Survey	402	NA	25-34
[48]	2013	Journal	Computers in Human Behavior	Survey	344	166 males, 178 females	12-17
[49]	2012	Journal	Computers in Human Behavior	Survey	749	NA	NA
[50]	2013	Journal	Information & Management	Survey	Total: 225 (USA:103, Taiwan:122)	USA: 64 males & 39 females, Taiwan: 53 males & 69 females	NA
[51]	2013	Journal	Computers in Human Behavior	Survey	368	171 males, 197 females	19-25 years
[52]	2011	Journal	Computers in Human Behavior	Survey	126 university students	36 males, 91 females.	NA
[53]	2012	Journal	Computers in Human Behavior	Experiment	Experiment 1: 79 undergraduates, Experiment 2: 72 undergraduates	Experiment 1: 20 males & 58 females,.Experiment 2:27 males & 45 females	18 22 years
[54]	2012	Journal	Computers in Human Behavior	Survey	238	135 males & 103 females	Mean 19 years
[55]	2013	Journal	Computers in Human Behavior	Survey	488 users	NA	NA
[56]	2013	Journal	Computers in Human Behavior	Survey	124 older adults	NA	60 90 years
[57]	2014	Journal	Computers in Human Behavior	Survey	315	60% males, 40% females	20 29 years.
[58]	2013	Journal	International Journal of Human-Computer Studies	Interview, Experiment	46	NA	NA
[59]	2011	Journal	Computers in Human Behavior	Survey	267	65% male, 35% females	Average 20 years
[60]	2013	Journal	Computers in Human Behavior	Survey	382	39.2% males 60.8% females	Mean 30.68 years

Table E. Details of Primary Study Publication Type, Year of Publication, Research Method, Participants and Duration

Paper Id	Year of Publication	Publication Type	Conference/Journal Name	Research Type	No. of Participants	Gender	Age
[61]	2013	Journal	Decision Support Systems	Survey	222	60% males, 40% females	Average 20 years
[62]	2013	Journal	Decision Support Systems	Survey	222	60% males, 40% females	Average 20 years
[63]	2012	Journal	Computers in Human Behavior	Survey	300	97 males, 207 females.	NA
[64]	2012	Journal	Computers in Human Behavior	Survey	148 students	54.7% males, 45.3% females	NA
[65]	2010	Journal	Computers in Human Behavior	Survey	959	33% males and 67% females	NA
[66]	2013	Journal	Decision Support Systems	Survey	458 facebook users	47.9% males, 52.1% females	18 - 24 years
[67]	2011	Journal	Computers in Human Behavior	Survey	122 undergraduates	40% males, 60% females	18 - 23 years.
[68]	2013	Journal	Computers in Human Behavior	Survey	271	48.3% males, 51.7% females	20-29 years
[69]	2013	Journal	Computers in Human Behavior	Survey	439	NA	16 45 years
[70]	2013	Journal	Computers in Human Behavior	Survey	1st Study:344 students, 2nd Study: 274 students	1st Study:62 males & 282 females, 2nd Study:232 female & 42 male	25.87 years
[71]	2013	Journal	Computers in Human Behavior	Survey	China: 400 students, USA: 490 students	China:27% males, 73% females, USA:31% males, 69% females.	NA
[72]	2011	Journal	Computers in Human Behavior	Survey	113 undergraduate	48 males, 65 females	18 - 36 years
[73]	2013	Journal	Computers in Human Behavior	Survey	201 college students	123 women, 77 male	1729 years

Table F. Details of Primary Study Publication Type, Year of Publication, Research Method, Participants and Duration

Paper Id	Year of Publication	Publication Type	Conference/Journal Name	Research Type	No. of Participants	Gender	Age
[74]	2013	Journal	Computers in Human Behavior	Survey	411	49.4% males, 50.6% females	NA
[75]	2013	Journal	Computers in Human Behavior	Survey	1st Study: 150 students, 2nd Study:317 students	1st study:51% females, 2nd Study:120 males,197 females	Average 20 years
[76]	2012	Journal	Computers in Human Behavior	Survey	463 students	29% males, 71% female	18-51 years
[77]	2012	Conference	IEEE 5th International Conference on New Technologies, Mobility and Security (NTMS)	Experiment Ex	600 friendship requests sent to random people on SNS.	NA	NA
[78]	2013	Conference	IEEE 10th International Conference on Service Systems and Service Management (ICSSSM)	Survey	118	NA	NA
[79]	2010	Conference	IEEE 43rd Hawaii International Conference on System Sciences (HICSS)	Survey	452 young adults	40% males,60% females	Average age 20.3 years.
[80]	2011	Conference	IEEE International Conference on Control System, Computing and Engineering (ICCSCE)	Survey	66 users	45.5% males, 54.5% females	21- 25 years.
[81]	2010	Conference	IEEE 43rd Hawaii International Conference on System Sciences (HICSS)	Survey	Germany: 138 users, USA: 193 users	Germany: 40.6% females, USA: 34.2% males & 65.3% females	18-29 years
[82]	2011	Conference	IEEE International Conference on Research and Innovation in Information Systems (ICRIIS)	Survey	340	58.8% females, 41.2% Males	NA
[83]	2011	Conference	IEEE International Conference on Research and Innovation in Information Systems (ICRIIS)	Survey	40 postgraduate students	NA	NA
[84]	2010	Conference	International Conference on Information and Communication Technology for the Muslim World (ICT4M), IEEE.	Survey	90 undergraduate and postgraduate students	NA	55.6% less than 20 years, 37.8% 21 to 25 years.

Table G. Details of Primary Study Publication Type, Year of Publication, Research Method, Participants and Duration

Paper Id	Year of Publication	Publication Type	Conference/Journal Name	Research Type	No. of Participants	Gender	Age
[85]	2012	Conference	International Conference on Technology Enhanced Education (ICTEE), IEEE.	Survey	145 students	NA	NA
[86]	2009	Conference	IEEE International Conference on Management of e-Commerce and e-Government, ICMECG'09.	Survey	351 users for survey,120 profiles chosen for analysis	NA	18-25 years
[87]	2009	Conference	3rd International Conference on Emerging Security Information, Systems and Technologies, SECURWARE, IEEE.	Experiment	19	NA	NA
[88]	2013	Conference	46th Hawaii International Conference on System Sciences (HICSS), IEEE.	Survey	322 users,161 responses from each country i.e. Singapore and America.	36.3% males, 63.7% females.	NA
[89]	2011	Conference	Proceedings of the 44th Hawaii International Conference on System Sciences, IEEE.	Interview	9 photographers	NA	25-35 years
[90]	2011	Conference	15th Panhellenic Conference on Informatics (PCI), IEEE.	Survey	144 teenager students	54 males, 46 females	16-17 years
[91]	2009	Conference	International Workshop on Social Informatics, IEEE.	Interview	11 employees	9 males, 2 females	25 55 years
[92]	2011	Conference	IEEE 3rd International Conference on Communication Software and Networks (ICCSN).	Survey	NA	NA	NA
[93]	2010	Conference	43rd Hawaii International Conference on System Sciences (HICSS), IEEE.	Survey	125	43.2% males, 56.8% females	19 28 years
[94]	2011	Conference	International Conference on Management and Service Science (MASS), IEEE	Survey	194	50% males, 50% females	20 to 24 years
[95]	2012	Conference	IEEE Transactions on Engineering Management.	Survey	367 students	41% females	18-24 years
[96]	2013	Conference	Fifth International Conference on Service Science and Innovation (ICSSI), IEEE	Survey	367 students	NA	NA
[97]	2010	Conference	IEEE 2nd Symposium on Web Society (SWS)	Survey	204	51.96% males, 48.04% females	

Table H. Details of Primary Study Publication Type, Year of Publication, Research Method, Participants and Duration

Paper Id	Year of Publication	Publication Type	Conference/Journal Name	Research Type	No. of Participants	Gender	Age
[98]	2011	Conference	44th Hawaii International Conference on System Sciences (HICSS), IEEE	Survey	325 undergraduates	Male 35.1% , Female 64.6%	Average Age 20.7 years.
[99]	2009	Conference	International Conference on Ultra Modern Telecommunications & Workshops ICUMT IEEE	Survey	934 students	60.6% Males,39.4% Females	18 to 23 years.
[100]	2010	Conference	IEEE Second International Conference on Social Computing (SocialCom)	Survey	113	NA	NA
[101]	2013	Conference	46th Hawaii International Conference on System Sciences (HICSS), IEEE	Interview	3 cases	NA	NA
[102]	2011	Conference	International Conference on Computer Science and Service System *CSSS*, IEEE	Survey	206 students	75 males, 128 females	Average age 19.3 years.
[103]	2011	Conference	2nd International Conference on Artificial Intelligence, Management Science and Electronic Commerce (AIMSEC), IEEE	Survey	140		
[104]	2012	Conference	45th Hawaii International Conference on System Science (HICSS), IEEE	Survey	200	48% males, 52% females	16-25 years.
[105]	2011	Conference	6th IEEE International Conference on Networking, Architecture and Storage (NAS), IEEE	Survey	266	NA	16 25 years.
[106]	2013	Conference	46th Hawaii International Conference on System Sciences (HICSS), IEEE	Survey	169 undergraduate students	38.5% males, 61.5% females	NA
[107]	2012	Conference	Information Security for South Africa *ISSA*, IEEE	Survey	100 users in surevy, 131 observed	75% males, 25% females	1830 years.
[108]	2011	Conference	IEEE International Conferences on Internet of Things, and Cyber, Physical and Social Computing	Experiment	500	125 young males, 125 young females, 125 older males and 125 older females	21 62 years
[109]	2011	Conference	International Conference on Advances in Social Networks Analysis and Mining	Survey	400 students	51.6%males, 48.4% females	20 or 21 years

Table I. Details of Primary Study Publication Type, Year of Publication, Research Method, Participants and Duration

Paper Id	Year of Publication	Publication Type	Conference/Journal Name	Research Type	No. of Participants	Gender	Age
[110]	2010	Conference	Seventh International Conference on Information Technology, IEEE	Survey	105	49 males, 556 females	21 27 years
[111]	2013	Conference	10th International Conference on Service Systems and Service Management (ICSSSM), IEEE	Survey	302	43% male,57% females	NA
[112]	2013	Conference	International Conference on Availability, Reliability and Security, IEEE	Survey	1024	52.1% male, 47.9% female	2029
[113]	2012	Conference	45th Hawaii International Conference on System Sciences, IEEE	Survey	313	69.6% female	Average age 21.67 years
[114]	2012	Conference	IEEE International Conference on Privacy, Security, Risk and Trust (PASSAT) and Social Computing (SocialCom)	Survey	100	males 75%, females 25%	21 27 years.
[115]	2011	Conference	IEEE Third International Conference on Privacy, Security, Risk and Trust (PASSAT) and Social Computing (SocialCom)	Survey	116	French users:24 male, 32 female. Hong Kong users: 16 male, 44 female	1740 years
[116]	2013	Conference	IEEE 3rd International Advance Computing Conference (IACC)	Survey	246 Facebook users	77.1% male, 22.9% female	16-35

References

1. Reynolds B, Venkatanathan J, Gonçalves J, Kostakos V. Sharing Ephemeral Information in Online Social Networks: Privacy Perceptions and Behaviours. In: Proceedings of the 13th IFIP TC 13 International Conference on Human-computer Interaction - Volume Part III. Springer-Verlag; 2011. p. 204–215.

2. Faisal M, Alsumait A. Social Network Privacy and Trust Concerns. In: Proceedings of the 13th International Conference on Information Integration and Web-based Applications and Services. ACM; 2011. p. 416–419.

3. Lampinen A, Lehtinen V, Lehmuskallio A, Tamminen S. We'Re in It Together: Interpersonal Management of Disclosure in Social Network Services. In: Proceedings of the SIGCHI Conference on Human Factors in Computing Systems. CHI '11. ACM; 2011. p. 3217–3226.

4. Jansen BJ, Sobel K, Cook G. Being Networked and Being Engaged: The Impact of Social Networking on Ecommerce Information Behavior. In: Proceedings of the 2011 iConference. iConference '11. ACM; 2011. p. 130–136.

5. Patil S. Will You Be My Friend?: Responses to Friendship Requests from Strangers. In: Proceedings of the 2012 iConference. iConference '12. ACM; 2012. p. 634–635.

6. Baumer EPS, Adams P, Khovanskaya VD, Liao TC, Smith ME, Schwanda Sosik V, et al. Limiting, Leaving, and (Re)Lapsing: An Exploration of Facebook Non-use Practices and Experiences. In: Proceedings of the SIGCHI Conference on Human Factors in Computing Systems. ACM; 2013. p. 3257–3266.

7. Giannakos MN, Giotopoulos KK, Chorianopoulos K. In the Face (Book) of the Daily Routine. In: Proceedings of the 14th International Academic MindTrek Conference: Envisioning Future Media Environments. ACM; 2010. p. 153–157.

8. Blasbalg J, Cooney R, Fulton S. Defining and Exposing Privacy Issues with Social Media. J Comput Sci Coll. 2012;28(2):6–14.

9. Zhao C, Jiang G. Cultural Differences on Visual Self-presentation Through Social Networking Site Profile Images. In: Proceedings of the SIGCHI Conference on Human Factors in Computing Systems. ACM; 2011. p. 1129–1132.

10. DiMicco JM, Millen DR. Identity Management: Multiple Presentations of Self in Facebook. In: Proceedings of the 2007 International ACM Conference on Supporting Group Work. GROUP '07. ACM; 2007. p. 383–386.

11. Sleeper M, Balebako R, Das S, McConahy AL, Wiese J, Cranor LF. The Post That Wasn'T: Exploring Self-censorship on Facebook. In: Proceedings of the 2013 Conference on Computer Supported Cooperative Work. CSCW '13. ACM; 2013. p. 793–802.

12. Lawler JP, Molluzzo JC. A Survey of First-year College Student Perceptions of Privacy in Social Networking. J Comput Sci Coll. 2011;26(3):36–41.

13. Halevi T, Lewis J, Memon N. A Pilot Study of Cyber Security and Privacy Related Behavior and Personality Traits. In: 22nd international conference on World Wide Web companion; 2013. p. 737–744.

14. Joinson AN. Looking at, Looking Up or Keeping Up with People?: Motives and Use of Facebook. In: Proceedings of the SIGCHI Conference on Human Factors in Computing Systems. ACM; 2008. p. 1027–1036.

15. Zhao C, Hinds P, Gao G. How and to Whom People Share: The Role of Culture in Self-disclosure in Online Communities. In: Proceedings of the ACM 2012 Conference on Computer Supported Cooperative Work. ACM; 2012. p. 67–76.

16. YangWang, Norcie G, Cranor LF. Who is Concerned About What? A Study of American, Chinese and Indian Users' Privacy Concerns on Social Network Sites. In: Proceedings of the 4th International Conference on Trust and Trustworthy Computing. Springer-Verlag; 2011. p. 146–153.

17. Bazarova NN. Contents and Contexts: Disclosure Perceptions on Facebook. In: Proceedings of the ACM 2012 Conference on Computer Supported Cooperative Work. CSCW '12. ACM; 2012. p. 369–372.

18. Burke M, Marlow C, Lento T. Feed Me: Motivating Newcomer Contribution in Social Network Sites. In: Proceedings of the SIGCHI Conference on Human Factors in Computing Systems. CHI '09. ACM; 2009. p. 945–954.

19. Steinfield C, DiMicco JM, Ellison NB, Lampe C. Bowling Online: Social Networking and Social Capital Within the Organization. In: Proceedings of the Fourth International Conference on Communities and Technologies. ACM; 2009. p. 245–254.

20. Shankar R. Factors Influencing Online Social Interactions. In: Proceedings of the 4th International Conference on Online Communities and Social Computing. OCSC'11. Springer-Verlag; 2011. p. 355–362.

21. Wang Y, Norcie G, Komanduri S, Acquisti A, Leon PG, Cranor LF. "I Regretted the Minute I Pressed Share": A Qualitative Study of Regrets on Facebook. In: Proceedings of the Seventh Symposium on Usable Privacy and Security. ACM; 2011. p. 10:1–10:16.

22. Seshagiri S. Content Consumption and Exchange Among College Students: A Case Study from India. In: Proceedings of the 8th International Conference on Mobile and Ubiquitous Multimedia. ACM; 2009. p. 3:1–3:9.

23. Tang D, Chou T, Drucker N, Robertson A, Smith WC, Hancock JT. A Tale of Two Languages: Strategic Self-disclosure via Language Selection on Facebook. In: Proceedings of the ACM 2011 Conference on Computer Supported Cooperative Work. ACM; 2011. p. 387–390.

24. Hart J, Ridley C, Taher F, Corina Sas AD. Exploring the Facebook Experience: A New Approach to Usability. In: Proceedings of the 5th Nordic Conference on Human-computer Interaction: Building Bridges. NordiCHI '08. ACM; 2008. p. 471–474.

25. Shi Y, Yue X, He J. Understanding Social Network Sites (SNSs) Preferences: Personality, Motivation, and Happiness Matters. In: Proceedings of the 5th International Conference on Online Communities and Social Computing. Springer-Verlag; 2013. p. 94–103.

26. Wisniewski P, Lipford H, Wilson D. Fighting for My Space: Coping Mechanisms for Sns Boundary Regulation. In: Proceedings of the SIGCHI Conference on Human Factors in Computing Systems. CHI '12. ACM; 2012. p. 609–618.

27. Shipps B, Phillips B. Social Networks, Interactivity and Satisfaction: Assessing Socio-technical Behavioral Factors As an Extension to Technology Acceptance. J Theor Appl Electron Commer Res. 2013;8(1):35–52.

28. Gray R, Ellison NB, Vitak J, Lampe C. Who Wants to Know?: Question-asking and Answering Practices Among Facebook Users. In: Proceedings of the 2013 Conference on Computer Supported Cooperative Work. ACM; 2013. p. 1213–1224.

29. Thomas L, Briggs P, Little L. Location Tracking via Social Networking Sites. In: Proceedings of the 5th Annual ACM Web Science Conference. ACM; 2013. p. 405–412.

30. Qiu L, Lin H, Leung AKy. How Does Facebook Browsing Affect Self-awareness and Social Well-being: The Role of Narcissism. In: Proceedings of the 7th International Conference on Advances in Computer Entertainment Technology. ACE '10. ACM; 2010. p. 100–101.

31. Wu J, Fu Z. Studying on the Using Patterns of Chinese SNS Platforms. In: Proceedings of the 2011 ACM Symposium on The Role of Design in UbiComp Research & Practice. ACM; 2011. p. 37–40.

32. Binder J, Howes A, Sutcliffe A. The Problem of Conflicting Social Spheres: Effects of Network Structure on Experienced Tension in Social Network Sites. In: Proceedings of the SIGCHI Conference on Human Factors in Computing Systems. CHI '09. ACM; 2009. p. 965–974.

33. Strater K, Lipford HR. Strategies and Struggles with Privacy in an Online Social Networking Community. In: Proceedings of the 22Nd British HCI Group Annual Conference on People and Computers: Culture, Creativity, Interaction - Volume 1. BCS-HCI '08. British Computer Society; 2008. p. 111–119.

34. Choi JH, Scott JE. Electronic Word of Mouth and Knowledge Sharing on Social Network Sites: A Social Capital Perspective. J Theor Appl Electron Commer Res. 2013;8(1):69–82.

35. Liu Y, Gummadi KP, Krishnamurthy B, Mislove A. Analyzing Facebook Privacy Settings: User Expectations vs. Reality. In: Proceedings of the 2011 ACM SIGCOMM Conference on Internet Measurement Conference. ACM; 2011. p. 61–70.

36. Pornsakulvanicha V, Dumrongsirib N. Internal and external influences on social networking site usage in Thailand. Computers in Human Behavior. 2013;29(6):2788 – 2795.

37. Wang JL, Jackson LA, Zhang DJ, Su ZQ. The Relationships Among the Big Five Personality Factors, Self-esteem, Narcissism, and Sensation-seeking to Chinese University Students' Uses of Social Networking Sites (SNSs). Comput Hum Behav. 2012;28(6):2313–2319.

38. Pai P, Arnott DC. User adoption of social networking sites: Eliciting uses and gratifications through a meansend approach. Computers in Human Behavior. 2013;29(3):1039 – 1053.

39. Ryan T, Xenos S. Who uses Facebook? An investigation into the relationship between the Big Five, shyness, narcissism, loneliness, and Facebook usage. Computers in Human Behavior. 2011;27(5):1658 – 1664.

40. Hum NJ, Chamberlin PE, Hambright BL, Portwood AC, Schat AC, Bevan JL. A picture is worth a thousand words: A content analysis of Facebook profile photographs. Computers in Human Behavior. 2011;27(5):1828 – 1833.

41. Mohamed N, Ahmad IH. Information privacy concerns, antecedents and privacy measure use in social networking sites: Evidence from Malaysia. Computers in Human Behavior. 2012;28(6):2366 – 2375.

42. Ross C, Orr ES, Sisic M, Arseneault JM, Simmering MG, Orr RR. Personality and motivations associated with Facebook use. Computers in Human Behavior. 2009;25(2):578 – 586.

43. Chang YP, Zhu DH. The role of perceived social capital and flow experience in building users continuance intention to social networking sites in China. Computers in Human Behavior. 2012;28(3):995 – 1001.

44. Chang YP, Zhu DH. Understanding social networking sites adoption in China: A comparison of pre-adoption and post-adoption. Computers in Human Behavior. 2011;27(5):1840 – 1848.

45. Panek ET, Nardis Y, Konrath S. Mirror or Megaphone?: How relationships between narcissism and social networking site use differ on Facebook and Twitter. Computers in Human Behavior. 2013;29(5):2004 – 2012.

46. Baker RK, White KM. Predicting Adolescents' Use of Social Networking Sites from an Extended Theory of Planned Behaviour Perspective. Comput Hum Behav. 2010;26(6):1591–1597.

47. Lin KY, Lu HP. Why people use social networking sites: An empirical study integrating network externalities and motivation theory. Computers in Human Behavior. 2011;27(3):1152 – 1161.

48. Apaolaza V, Hartmann P, Medina E, Barrutia JM, Echebarria C. The relationship between socializing on the Spanish online networking site Tuenti and teenagers subjective wellbeing: The roles of self-esteem and loneliness. Computers in Human Behavior. 2013;29(4):1282 – 1289.

49. Omoush KSA, Yaseen SG, Almaaitah MA. The impact of Arab cultural values on online social networking: The case of Facebook. Computers in Human Behavior. 2012;28(6):2387 – 2399.

50. Ku YC, Chen R, Zhang H. Why do users continue using social networking sites? An exploratory study of members in the United States and Taiwan. Information & Management. 2013;50(7):571 – 581.

51. Lee DY. The role of attachment style in building social capital from a social networking site: The interplay of anxiety and avoidance. Computers in Human Behavior. 2013;29(4):1499 – 1509.

52. Tokunaga RS. Social networking site or social surveillance site? Understanding the use of interpersonal electronic surveillance in romantic relationships. Computers in Human Behavior. 2011;27(2):705 – 713.

53. Gentile B, Twenge JM, Freeman EC, Campbell WK. The effect of social networking websites on positive self-views: An experimental investigation. Computers in Human Behavior. 2012;28(5):1929 – 1933.

54. Muscanell NL, Guadagno RE. Make new friends or keep the old: Gender and personality differences in social networking use. Computers in Human Behavior. 2012;28(1):107 – 112.

55. Trepte S, Reinecke L. The reciprocal effects of social network site use and the disposition for self-disclosure: A longitudinal study. Computers in Human Behavior. 2013;29(3):1102 – 1112.

56. Braun MT. Obstacles to social networking website use among older adults. Computers in Human Behavior. 2013;29(3):673 – 680.

57. Lu HP, Yang YW. Toward an understanding of the behavioral intention to use a social networking site: An extension of task-technology fit to social-technology fit. Computers in Human Behavior. 2014;34(0):323 – 332.

58. Lee H, Park H, Kim J. Why do people share their context information on Social Network Services? A qualitative study and an experimental study on users' behavior of balancing perceived benefit and risk. International Journal of Human-Computer Studies. 2013;71:862 – 877.

59. Smock AD, Ellison NB, Lampe C, Wohn DY. Facebook as a toolkit: A uses and gratification approach to unbundling feature use. Computers in Human Behavior. 2011;27(6):2322 – 2329.

60. Hossain MD, Veenstra AS. Online maintenance of life domains: Uses of social network sites during graduate education among the {US} and international students. Computers in Human Behavior. 2013;29(6):2697 – 2702.

61. Chen R. Living a private life in public social networks: An exploration of member self-disclosure. Decision Support Systems. 2013;55(3):661 – 668.

62. Chen R. Member use of social networking sites an empirical examination. Decision Support Systems. 2013;54(3):1219 – 1227.

63. Hughes DJ, Rowe M, Batey M, Lee A. A tale of two sites: Twitter vs. Facebook and the personality predictors of social media usage. Computers in Human Behavior. 2012;28(2):561 – 569.

64. Xu C, Ryan S, Prybutok V, Wen C. It is not for fun: An examination of social network site usage. Information & Management. 2012;49(5):210 – 217.

65. Correa T, Hinsley AW, de Zúñiga HG. Who interacts on the Web?: The intersection of users' personality and social media use. Computers in Human Behavior. 2010;26(2):247 – 253.

66. Al-Debei MM, Al-Lozi E, Papazafeiropoulou A. Why people keep coming back to Facebook: Explaining and predicting continuance participation from an extended theory of planned behaviour perspective. Decision Support Systems. 2013;55(1):43 – 54.

67. Stutzman F, Capra R, Thompson J. Factors mediating disclosure in social network sites. Computers in Human Behavior. 2011;27:590 – 598.

68. Pi SM, Chou CH, Liao HL. A study of Facebook Groups members knowledge sharing. Computers in Human Behavior. 2013;29(5):1971 – 1979.

69. Ku YC, Chu TH, Tseng CH. Gratifications for using {CMC} technologies: A comparison among SNS, IM, and e-mail. Computers in Human Behavior. 2013;29(1):226 – 234.

70. Grieve R, Indian M, Witteveen K, Anne Tolan G, Marrington J. Face-to-face or Facebook: Can Social Connectedness Be Derived Online? Comput Hum Behav. 2013;29(3):604–609.

71. Jackson LA, Wang JL. Cultural differences in social networking site use: A comparative study of China and the United States. Computers in Human Behavior. 2013;29(3):910 – 921.

72. Underwood JDM, Kerlin L, Farrington-Flint L. The lies we tell and what they say about us: Using behavioural characteristics to explain Facebook activity. Computers in Human Behavior. 2011;27(5):1621 – 1626.

73. Tazghini S, Siedlecki KL. A mixed method approach to examining Facebook use and its relationship to self-esteem. Computers in Human Behavior. 2013;29(3):827 – 832.

74. Rui J, Stefanone MA. Strategic self-presentation online: A cross-cultural study. Computers in Human Behavior. 2013;29(1):110 – 118.

75. Sheldon P. Voices that cannot be heard: Can shyness explain how we communicate on Facebook versus face-to-face? Computers in Human Behavior. 2013;29(4):1402 – 1407.

76. Chen B, Marcus J. Students self-presentation on Facebook: An examination of personality and self-construal factors. Computers in Human Behavior. 2012;28(6):2091 – 2099.

77. Jabeur N, Zeadally S, Maydebura S. Improving Trust and Privacy Models in Social Networks. In: New Technologies, Mobility and Security (NTMS), 2012 5th International Conference on; 2012. p. 1–5.

78. Huang CH, Hsu MC. Acceptance of location-based service technology x2014;The Facebook Check-in function. In: Service Systems and Service Management (ICSSSM), 2013 10th International Conference on; 2013. p. 809–812.

79. Rosen D, Stefanone MA, Lackaff D. Online and Offline Social Networks: Investigating Culturally-specific Behavior and Satisfaction. In: System Sciences (HICSS), 2010 43rd Hawaii International Conference on. IEEE; 2010. p. 1–10.

80. Almadhoun NM, Dominic PDD, Woon LF. Perceived security, privacy, and trust concerns within Social Networking Sites: The role of Information sharing and relationships development in the Malaysian Higher Education Institutions' marketing. In: Control System, Computing and Engineering (ICCSCE), 2011 IEEE International Conference on; 2011. p. 426–431.

81. Krasnova H, Veltri NF. Privacy Calculus on Social Networking Sites: Explorative Evidence from Germany and USA. In: System Sciences (HICSS), 2010 43rd Hawaii International Conference on; 2010. p. 1–10.

82. Mohamed N, Ahmad IH. Privacy measures awareness, privacy setting use and information privacy concern with Social Networking Sites. In: Research and Innovation in Information Systems (ICRIIS), 2011 International Conference on; 2011. p. 1–6.

83. Osman FY, Rahim NZA. Self-disclosure and Social network sites users' awareness. In: Research and Innovation in Information Systems (ICRIIS), 2011 International Conference on; 2011. p. 1–6.

84. Zamzami IF, Olowolayemo A, Bakare KK, Kind DA. Sensitivity to online privacy in social networking sites. In: Information and Communication Technology for the Muslim World (ICT4M), 2010 International Conference on; 2010. p. B–21–B–26.

85. Dhume SM, Pattanshetti MY, Kamble SS, Prasad T. Adoption of social media by Business Education students: Application of Technology Acceptance Model (TAM). In: Technology Enhanced Education (ICTEE), 2012 IEEE International Conference on; 2012. p. 1–10.

86. Lang M, Devitt J, Kelly S, Kinneen A, OMalley J, Prunty D. Social Networking and Personal Data Security: A Study of Attitudes and Public Awareness in Ireland. In: Management of e-Commerce and e-Government, 2009. ICMECG '09. International Conference on; 2009. p. 486–490.

87. Nagy J, Pecho P. Social Networks Security. In: Emerging Security Information, Systems and Technologies, 2009. SECURWARE '09. Third International Conference on; 2009. p. 321–325.

88. Rui J, Stefanone MA. Strategic Management of Other-Provided Information Online: Personality and Network Variables. In: System Sciences (HICSS), 2013 46th Hawaii International Conference on; 2013. p. 3941–3950.

89. Malinen S. Strategies for Gaining Visibility on Flickr. In: System Sciences (HICSS), 2011 44th Hawaii International Conference on; 2011. p. 1–9.

90. Faliagka E, Tsakalidis A, Vaikousi D. Teenagers' Use of Social Network Websites and Privacy Concerns: A Survey. In: Informatics (PCI), 2011 15th Panhellenic Conference on; 2011. p. 207–211.

91. Olsen LE, Guribye F. The Adoption of Social Networking Services. Social Informatics, International Workshop on. 2009;0:3–9.

92. Shin S, Ko Y, Jang J. The conflict between privacy and self-disclosure in Social Networking Services. In: Communication Software and Networks (ICCSN), 2011 IEEE 3rd International Conference on; 2011. p. 490–493.

93. Shi N, Lee MKO, Cheung C, Chen H. The Continuance of Online Social Networks: How to Keep People Using Facebook? In: System Sciences (HICSS), 2010 43rd Hawaii International Conference on; 2010. p. 1–10.

94. Qi Y, Fu C. The Effects of Flow and Attachment on the e-Loyalty of SNS Websites. In: International Conference on Management and Service Science (MASS); 2011. p. 1–6.

95. Lankton NK, McKnight DH, Thatcher JB. The Moderating Effects of Privacy Restrictiveness and Experience on Trusting Beliefs and Habit: An Empirical Test of Intention to Continue Using a Social Networking Website. Engineering Management, IEEE Transactions on. 2012;59(4):654–665.

96. Lin KM. The Negative Critical Incidents of Social Network Service: An Exploratory Study. In: Service Science and Innovation (ICSSI), 2013 Fifth International Conference on; 2013. p. 96–99.

97. Yu L, Wu M. The relation of personality and self-disclosure on Renren. In: Web Society (SWS), 2010 IEEE 2nd Symposium on; 2010. p. 435–442.

98. Vitak J, Ellison NB, Steinfield C. The Ties That Bond: Re-Examining the Relationship between Facebook Use and Bonding Social Capital. In: System Sciences (HICSS), 2011 44th Hawaii International Conference on; 2011. p. 1–10.

99. Stanoevska-Slabeva K, Stanoevska-Slabeva K, Wozniak T, Hoffend I, Ebermann J. Towards a concept for inclusion of social network information as context information. In: Ultra Modern Telecommunications Workshops, 2009. ICUMT '09. International Conference on; 2009. p. 1–5.

100. Ngeno C, Zavarsky P, Lindskog D, Ruhl R. User's Perspective: Privacy and Security of Information on Social Networks. In: Social Computing (SocialCom), 2010 IEEE Second International Conference on; 2010. p. 1038–1043.

101. Desiato C. Virtualizing the Past: Re-connecting on Facebook and Emerging Social Relationships. In: System Sciences (HICSS), 2013 46th Hawaii International Conference on; 2013. p. 3363–3372.

102. Cao Z, Lin Y, Zhao C. An empirical study of user's attitudes and behavior of privacy concerns. In: Computer Science and Service System (CSSS), 2011 International Conference on; 2011. p. 2029–2034.

103. Jiang X, Du R, Ai SZ. An empirical study of users' continued usage of social networking service website. In: Artificial Intelligence, Management Science and Electronic Commerce (AIMSEC), 2011 2nd International Conference on; 2011. p. 105–108.

104. Lee JER, Moore DC, Park EA, Park SG. Who wants to be friend-rich? Social compensatory friending on Facebook and the moderating role of public self-consciousness. Computers in Human Behavior. 2012;28(3):1036 – 1043.

105. Bao Y, Wang X, Deng D. Applying Modified TAM to Privacy Setting Tools on SNS. In: Networking, Architecture and Storage (NAS), 2011 6th IEEE International Conference on; 2011. p. 40–44.

106. Lin X, Li Y, Califf CB, Featherman M. Can Social Role Theory Explain Gender Differences in Facebook Usage? In: System Sciences (HICSS), 2013 46th Hawaii International Conference on; 2013. p. 690–699.

107. Ntlatywa P, Botha RA, Haskins B. Claimed vs observed information disclosure on social networking sites. In: Information Security for South Africa (ISSA). IEEE; 2012. p. 1–6.

108. Quinn D, Chen L, Mulvenna M. Does Age Make a Difference in the Behaviour of Online Social Network Users? In: Internet of Things (iThings/CPSCom), 2011 International Conference on and 4th International Conference on Cyber, Physical and Social Computing; 2011. p. 266–272.

109. Uesugi S. Effects of Personality Traits on Usage of Social Networking Service. In: Advances in Social Networks Analysis and Mining (ASONAM), 2011 International Conference on; 2011. p. 629–634.

110. Jia Y, Zhao Y, Lin Y. Effects of System Characteristics on Users' Self-Disclosure in Social Networking Sites. In: Information Technology: New Generations (ITNG), 2010 Seventh International Conference on; 2010. p. 529–533.

111. Song Z, Hao C, Daqing Z. Empirical study on users' participation behavior in SNS based on theory of perceived risks and involvement degree. In: Service Systems and Service Management (ICSSSM), 2013 10th International Conference on; 2013. p. 424–429.

112. Otsuki M, Sonehara N. Estimating the Value of Personal Information with SNS Utility. In: Availability, Reliability and Security (ARES), 2013 Eighth International Conference on; 2013. p. 512–516.

113. Marder B, Joinson A, Shankar A. Every Post You Make, Every Pic You Take, I'll Be Watching You: Behind Social Spheres on Facebook. In: System Science (HICSS), 2012 45th Hawaii International Conference on; 2012. p. 859–868.

114. Ntlatywa P, Botha RA, Haskins B. Factors that Influence the Choice of Privacy Settings on Facebook: Freshmen's View at a South African University. In: Privacy, Security, Risk and Trust (PASSAT), 2012 International Conference on and 2012 International Confernece on Social Computing (SocialCom); 2012. p. 843–850.

115. Tsoi HK, Chen L. From Privacy Concern to Uses of Social Network Sites: A Cultural Comparison via User Survey. In: Privacy, Security, Risk and Trust (PASSAT) and 2011 IEEE Third Inernational Conference on Social Computing (SocialCom), 2011 IEEE Third International Conference on; 2011. p. 457–464.

116. Dhami A, Agarwal N, Chakraborty TK, Singh BP, Minj J. Impact of trust, security and privacy concerns in social networking: An exploratory study to understand the pattern of information revelation in Facebook. In: Advance Computing Conference (IACC), 2013 IEEE 3rd International; 2013. p. 465–469.
